# Is There a Sex Difference in Technical Skills among Youth Soccer Players in Norway?

**DOI:** 10.3390/sports10040050

**Published:** 2022-03-29

**Authors:** Arne Sørensen, Emma C. Haugen, Roland van den Tillaar

**Affiliations:** Department of Sport Sciences and Physical Education, Nord University, 7600 Levanger, Norway; emma.c.haugen@student.nord.no (E.C.H.); roland.v.tillaar@nord.no (R.v.d.T.)

**Keywords:** soccer, technical skills, technical test, long passes, reception

## Abstract

Female soccer has recently experienced an impressive increase in the number of players, and an impressive improvement in the quality of elite matches. Still, studies show sex differences in match statistics on passing accuracy and the ability to control the ball in international matches, which is explained by a lower skill of level in female soccer players as compared to male players. Therefore, the aim of this study was to evaluate if female youth soccer players had bridged the gap in technical skills to reach the level that boys have traditionally attained. Sixteen male and 17 female youth soccer players of the same age and experience level took part in technical skill tests of reception of the ball on the ground and long passes. The results show a significant difference between the sexes in reception performance in favour of the male players (*p* < 0.05, ES = 1.09), but no significant difference in the long pass test (*p* = 0.11, ES = 0.43). This leads to the conclusion that the lower score on ball reception is probably the result of experience in small-sided self-organised soccer games during childhood among the male players, which influences reception skills but not the ability to make accurate long passes.

## 1. Introduction

Organised female soccer started in several European countries in the mid-1960s, and it has continued to experience an impressive increase in participants. The latest information from Federation Internationale de Football Association (FIFA) shows that worldwide around 30 million female senior and youth players take part in a football club [1]. Even though the quality of female soccer is improving, media interest and the economy is not in the same league as male soccer [2]. The critique of female soccer has often been about low pace and poor skills in comparison to male soccer [3]. Research has proven that there is a difference in performance between sexes in all sports with high demands for physical capacity [4,5].

High performance in soccer is determined by skill levels of players, with skills defined as a combination of technical and tactical ability in the rapidly changing environment of soccer games [6]. Analyses of the top matches of clubs and national teams for both sexes show a similarity with regard to the number of ball touches and time in possession of the ball [7]. Differences have also been detected, such as female players lost the ball more often [8], had a higher percentage of mistakes in reception and passes, and made more long passes from the defensive area [9]. Suggested reasons for sex differences in match performance are that female players experience more fatigue than male players during matches [7], that game tactics in female soccer do not focus as much on possession of the ball as do male game tactics and that female players make more long passes from the defensive area [9]. This has led to a lower number of passes and more passes with a higher possibility of interference. Finally, these differences could also mean that female players have lower technical and tactical soccer skills than male players.

Studies that have evaluated the sex differences in general motor control show that young men perform better in gross motor skills and young women are better at mastering activities which involve fine motor skills [10,11]. Furthermore, young men show significantly better performance than young women on tests of object control, such as throwing and kicking [12].

Differences in dribble speed have been found in youth soccer players [13,14], and it has been argued that much of the difference can be explained by sex difference in sprinting speed [13]. Analyses of the ability to rapidly change direction (COD), which is a vital part of dribbling speed, reveal relative strength to be decisive for high performance [15]. One study reported no significant sex differences among youth players in important technical soccer skills, such as long passes, heading and shooting [14], while differences in favour of male players in technical passing skills were found in another [16].

Little research on senior soccer has been published related to sex differences in soccer skills. Two separate studies have shown large differences in the Loughborough Soccer Passing Test [17,18] and dribble speed in favour of male players [19]. According to Thomas and French [20], the reason for these sex differences in motor control is more likely to be environmental than biological, as the superiority of boys in gross motor skills and differences in technical skills can probably be explained by the fact that males have done more of this type of motor skill training than have females.

The observed improvement in the quality of female international soccer and the increasing popularity for girls to start soccer training at an early age [1] leads to an interesting question: Have youth female soccer players bridged the gap in soccer skills to reach the level that boys have traditionally attained? Findings from previous studies on this question are unclear because most have used dribble speed as a sign of skill level. Since dribble speed is correlated with sprint speed [13] and COD which depend on muscular strength [15], these tests will favour male players. Therefore, it is important to bring new attention to the subject and evaluate other soccer skills in which the sex difference in physical strength is reduced. The ability to control the ball and make accurate passes has been shown to be different in elite matches for males and females [7,8]. Our purpose is, therefore, to examine if there is a sex difference in such skills as long passes and ball reception among youth soccer players. The hypothesis of this study was that youth female players are at the same technical level as youth male players.

## 2. Materials and Methods

### 2.1. Method

It is a challenge to design a relevant test to evaluate a possible sex difference in skill level in soccer because the quality of the opponent plays a decisive role in the performance in matches [21]. It is difficult to test soccer skills with high reliability and construct validity [22], and the measurement of decision making by means of an iPad [23] provides uncertain information on players’ tactical abilities. Therefore, we chose to test player technical levels to evaluate if there is a sex difference in the technical part of soccer skills. This design will not directly measure the complete skill level, but since level of technical ability in soccer is related to level of competition [24] and technical tests could provide high reliability [6], we decided to use those tests in this study.

### 2.2. Participants

Seventeen female outfield soccer players (age 17.8 ± 0.6 years; height 1.69 ± 0.05 m; body mass 62.9 ± 6.04 kg) and 16 male outfield soccer players (age 17.8 ± 0.7 years; height 1.78 ± 0.07 m; body mass 71.1 ± 6.7 kg) participated in the study. The inclusion criteria were that the participants were active players in soccer clubs, and the exclusion criteria were that they did not have any injuries or illness on the test days. All male players competed in teams in the first division of the regional U18 league, and female players competed in senior teams at the third and fourth levels. The tests were executed in November and December, where the soccer season was finished in the Norwegian soccer leagues. In this period, the players have a relatively low training load, which will not influence the skill results as much. The players signed a written consent form to take part in this study, in accordance with regulations of the Norwegian Centre for Research Data. Approval to use the data and to conduct the study was given by the Norwegian Centre for Research Data (reference code nr. 835109), on 13 August 2020.

### 2.3. Procedures

Two technique tests were conducted to evaluate if there was a sex difference in technical skills in passing and reception of the ball in youth soccer players. During the test of reception of the ball on the ground, player performances were recorded using an AV video camera (Sony PXW-Z90, Sony, Tokyo, Japan).

All tests were conducted in an indoor soccer hall with artificial turf, and similar balls (size 5) with identical air pressure were used (0.6 bar). The players took part in a general standardised warm-up (15 min) of running and dribbling with each ball (5 min) and passing and reception from different distances (10 min) before the test. Information about their soccer histories, daily training, match frequency, and level of match play was gathered on test day from the players.

**Figure 1:**
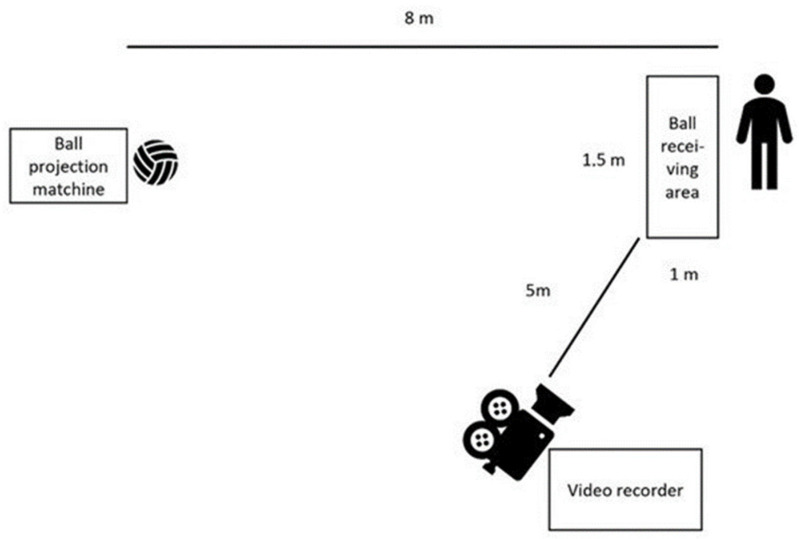
The set up for the ball reception test.

After the warm-up, the participants stood 8 m away from the ball projection machine (Sport Tutor, Burbank, CA, USA) and were instructed to receive the ball in the way they wanted for the purpose of controlling the ball inside a marked area in front of them (1 × 1.5 m, see Figure 1). The participants were instructed to make one touch in the reception and then one touch to pass the ball in the direction of the ball projection machine. The participants completed 10 attempts to receive the ball on the ground with a ball speed at 19 km/h (measured with a laser gun (Stalker Pro II+, Richardson, TX, USA)) and were instructed to make the receive every second time to the right and left to make a pass with the right or left foot. The test was adapted from [25] (Table 1). Two educated soccer coaches, one with a UEFA b-licence and with more than 10 years of experience as a soccer coach and one with a UEFA c-licence with more than 5 years of experience, scored every reception of the ball based on set criteria [25].

**Figure 2:**
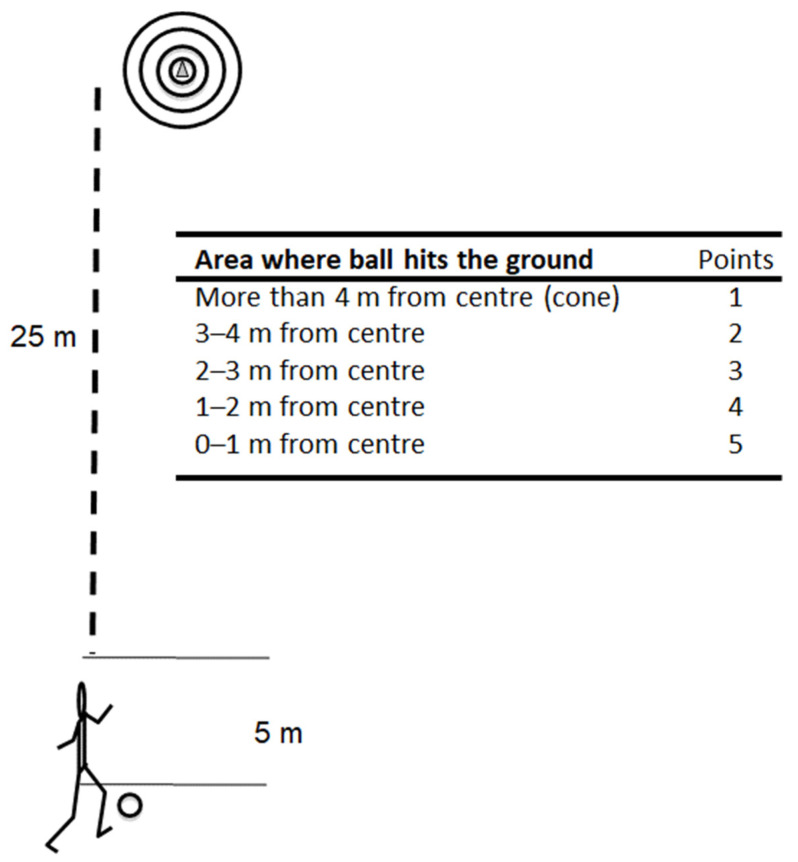
Test on long passes with criteria for scoring the test.

After the ball reception test, the participants started with the ball 30 m away from a target (a large cone) and were instructed to take one touch of the ball inside a 5 m area followed by a long pass in the air with their best foot (see Figure 2). The participants made the pass when the ball was rolling. The aim was to pass the ball through the air and hit the ground as close to the marker as possible. There were four circles with 2 m increase in diameter around the cone with the highest score closest to the cone and the lowest outside the largest circle (5-1 points; Figure 2), modified after [26]. Two judges were standing 5 m from the marker and scored each trial; if the ball bounced at the line, the highest score was given. The participants made two familiarisation trials, and then 15 trials in the test. Players’ average scores were used for further analysis. A relatively short distance was chosen (25 m) for the long pass test to minimise the effect of sex difference in strength on the results.

### 2.4. Statistical Analysis

Players’ average scores for each test were used for further analysis. The data were expressed as mean ± standard derivation (SD), and we also reported Cohen’s d effect size for sex differences in both technical tests. An effect size of 0.2 was considered small, 0.5 medium, and 0.8 large [27]. The difference between sexes was analysed by independent sample T-test with a significance level of *p* ≤ 0.05. A Pearson’s correlation was used for all participants and per sex to investigate correlations between the scores of the ball reception and long passes. Threshold values for the correlation coefficients’ interpretation as an effect size were 0.1–0.3 (trivial), 0.3–0.5 (moderate), 0.5–0.7 (large), and 0.7–0.9 (very large; Hopkins et al., 2009).

A repeatability analysis was performed on a subset of 50 randomly chosen samples and assessed at two different points in time by two coaches to evaluate the accuracy of the judgements of the test ratings. Mean estimates with 95% confidence intervals (CI) were reported for an intraclass correlation coefficient (ICC). Interpretation was as follows: <0.50 poor; from 0.50 to 0.75 fair; from 0.75 to 0.90 good; and above 0.90 excellent. The ICC for inter-rater reliability between coaches was good to excellent at 0.90. All statistical analyses were performed using SPSS Statistical Analysis Software for Windows^®^ (SPSS, version 25, Chicago, IL, USA).

## 3. Results

No significant differences were found between sexes for soccer experience and training experience per week (t ≤ 1.46, *p* ≥ 0.155, ES ≤ 0.5, Table 2).

**Figure 3:**
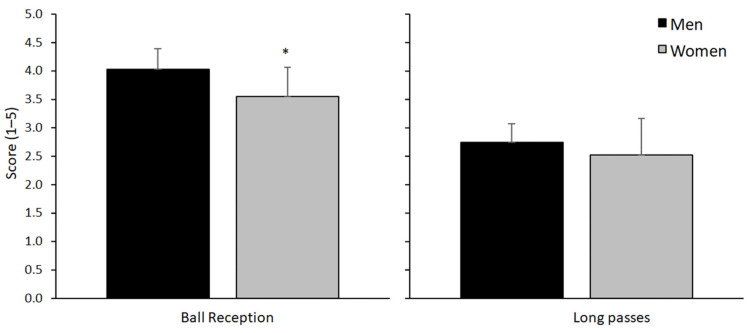
Mean (±SD) score for the ball reception and long passes tests for male and female youth soccer players; * indicates a significant difference between the sexes on a *p* ≤ 0.05 level.

A significant difference between the sexes was found in the ball reception test (t = 3.1, *p* = 0.004, ES = 1.09) but not in the long passes test (t= 1.2, *p* = 0.22, ES = 0.43, Figure 3). The individual scores for ball reception varied from 2.4 to 4.4 points, while the long passes’ scores varied from 1.53 to 3.8 (Figure 3).

**Figure 4:**
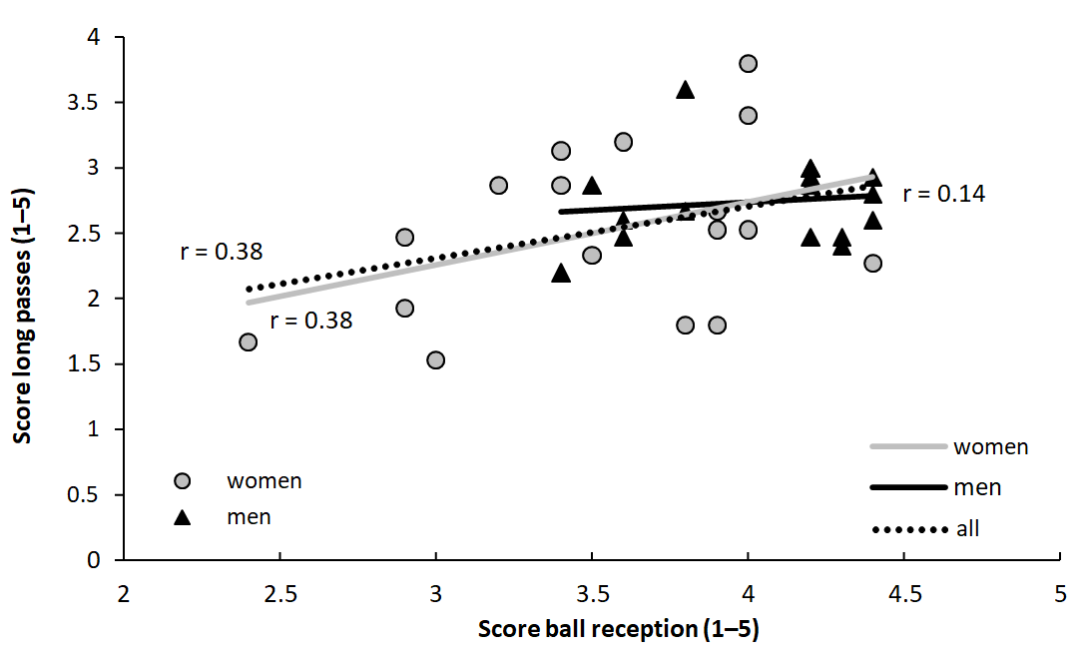
Correlation between ball reception and long passes tests for the whole group and specified per sex.

A significant moderate positive correlation (r = 0.38, *p* = 0.028) between the scores for the ball reception and long passes was found for the whole group, but when specified per men (r = 0.14, *p* = 0.61) and women (r = 0.38, *p* = 0.125), only trivial and moderate nonsignificant correlations were found (Figure 4).

## 4. Discussion

The aim of the study was to examine if there was a sex difference in the skills in long passes and reception of the ball in youth soccer players. The main findings were a significantly higher accuracy in reception of the ball on the ground for male youth players compared to female youth players, but not in long passes. Thereby, the hypothesis was partly correct. The participants in this study were equal in terms of age, years in a soccer club, and hours of soccer training in school and club, and both male and female players took part in one match per week during the season (Table 2). Theoretically, only environmental differences should explain the sex differences in the test [20]. The explanation of the findings could be that—as the authors of this study have experienced—far more boys than girls participate at a young age (6–12 years) in soccer play in school and in their spare time. This is a crucial period to improve skills in soccer, and research has shown that players who take a greater part in these activities increase their chances of becoming a professional soccer player (Ford et al., 2009).

Our finding of no sex difference in long passes was not in line with a study of Finish youth soccer players who showed significant differences in passing performance [16]. It is worth noting that these results are from surveys conducted between 2002 and 2010. The development of female soccer players’ skill levels has increased after that. Our findings of sex difference in accuracy in reception are in contrast to Perroni [14], who found no significant differences between sexes on tests of other technical skills in soccer such as passing, heading, and shooting for youth players. These authors also found that female players performed significantly better than males in one juggling test. This is rather surprising given that the male players in Perroni et al.’s study had been part of a football club for a longer period of time than the females and took part in more football practice each week.

We found no significant difference between sexes in the test of accuracy in long passes. This indicated that the distance of the long pass of 25 m did not discriminate against the female players with regard to their lower muscular strength. The reason for no significant sex difference could be the equality in their soccer history. Both sexes started with soccer when they were approximately 6 years old and have played on a soccer team for about 10 years.

The reason for the significant difference in performance of receptions but not for long passes could possibly be explained by the ‘early engagement hypothesis’ [28,29] and the specificity principle of training [30]. If both sexes practised long passes for an equal time, the improvement should be the same [20]. Better performance in reception by the male players indicates a large amount of training of the actual skill, probably through more soccer play in childhood. Both social and cultural reasons could explain the observed issue that boys take more part in unorganised soccer play in the period from 6 to 12 years [31]. There is reason to believe that soccer play in small groups (SSG) will have more influence on the ability of reception than on long passes [32] since such activities are often performed in a limited area (school yards) where the need to make long passes is low.

The individual results of reception (Figure 4) showed that one female player scored points equal to that of the best performing male players. Furthermore, a female player had the best performance in the test of long passes. This indicates that with enough training, female players could score just as well as male players in the technical tests used in the present study.

A moderate significant correlation between the two technical tests was found when the whole group performance was used while only trivial and moderate nonsignificant correlations for each sex were found. This shows that high quality performance in one technical skill in soccer does not automatically indicate high performance on another. These findings are in accordance with the theory about the transfer of motoric learning [33]. If players, for example, practice long passes, they will probably improve their performance, but this will not improve their ability to make accurate receptions. The reason is a low degree of similarity in the two skills [33].

The study has some limitations as the scoring system on both technical tests was from 1 to 5 points. A wider scoring system could have increased the sensitivity of the test to provide a better separation of player quality. A relatively limited number of attempts (10 and 15 in ball reception and long pass test, respectively) could have led to coincidences affecting the results. To ensure a higher reliability in tests, the quantity of attempts could have been higher [34]. Furthermore, it is possible that by adding more participants to the study, the results could have been different. In addition, the soccer level of participants could have affected the results, even though both groups were categorised as medium level players in the area. Care should be taken in generalisation of the results, since experience, level of competitiveness, and age can affect the technical level of players.

Future studies on this subject should evaluate technical skills in top soccer players, both youth players and adult players, to detect a possible sex difference. If there is a sex difference in technical skills so that male players perform better on technical tests, it would be reasonable to believe that the sex difference in match statistics would continue.

## 5. Conclusions

Youth female soccer players scored significantly lower on technical performance in reception but not in long passes. As reception is a vital skill in soccer, and poor quality will influence the opportunity to make a precise pass or a good shot. Thereby, the reason for sex differences in match statistics could be explained by differences in ability to make accurate receptions of the ball, which could influence the percentage of successful passing. The two groups of youth players had similar football history in a soccer club, but a hypothetical explanation of the finding could be more self-organised soccer play in childhood among the male players, as small-sided games influence reception skill rather than the ability to make accurate long passes. Thereby, sex differences still occur in ball reception.

## Figures and Tables

**Table 1:** The criteria for scoring the reception of the ball from the ball projection machine at a speed of 19 km/h from, adapted from [25].

Score	Criterium, Ball Reception, on the Ground, 19 km/t
1	The participant does not manage to stop the ball.
2	The participant loses control over the ball, and the ball goes outside the area in front of or beside him (1 × 1.5 m) before the player manages to pass the ball.
3	The participant manages to control the ball in the area, but the ball is not controlled in the correct direction (left or right).
4	The participant manages to control the ball in the area, in the correct direction (left or right), but is too close or too far from the player, resulting in difficulty in making the pass.
5	The participant manages to control the ball within the area, in the correct direction (left or right), and the receiving is perfect, so the pass is easy to perform.

**Table 2:** Participation in a soccer club, matches per week, and soccer training per week.

	Female Players	Male Players
Years in a soccer club	11 ± 1.3	11.2 ± 1.8
Matches per week	1	1
Soccer training in school and club (h/week)	8.8 ± 2.3	9.2 ± 0.9
Self-organised soccer training (h/week)	0.9 ± 0.9	1.5 ± 1.5

## Data Availability

The data presented in this study are available on request from the corresponding author. The data are not publicly available due to rules of Norwegian Center for Research Data.

## References

[1] (FIFA), F.I.d.F.A. Women’s Football Survey. https://www.icsspe.org/system/files/FIFA%20-%20Womens%20Football%20Survey.pdf.

[2] Hovden J., von der Lippe G. (2019). The gendering of media sport in the Nordic countries. Sport Soc..

[3] Hjelseth A., Hovden J. (2014). Negotiating the status of women’s football in Norway. An analysis of online supporter discourses. Eur. J. Sport Soc..

[4] Sandbakk Ø., Solli G.S., Holmberg H.-C. (2018). Sex Differences in World-Record Performance: The Influence of Sport Discipline and Competition Duration. Int. J. Sports Physiol. Perf..

[5] Thibault V., Guillaume M., Berthelot G., Helou N.E., Schaal K., Quinquis L., Nassif H., Tafflet M., Escolano S., Hermine O. (2010). Women and Men in Sport Performance: The Gender Gap has not Evolved since 1983. J. Sports Sci. Med..

[6] Ali A. (2011). Measuring soccer skill performance: A review. Scand. J. Med. Sci. Sports.

[7] Bradley P., Dellal A., Mohr M., Castellano J., Wilkie A. (2013). Gender differences in match performance characteristics of soccer players competing in the UEFA Champions League. Hum. Movem. Sci..

[8] Casal C., Losada J., Dios R., Arda A. (2020). Gender differences in technical-tactical behaviour of La Liga Spanish football teams. J. Hum. Sport Exerc..

[9] Hjelm J. (2011). The bad female football player: Women’s football in Sweden. Soccer Soc..

[10] Rodrigues P., Ribeiro M., Barros R., Lopes S., Sousa A. (2019). Performance on the movement assessment battery for children: A systematic review about gender differences. [Desempeño en la batería de evaluación del movimiento para niños: Una revisión sistemática sobre las diferencias de género]. Rev. Int. Cienc. Desporte.

[11] Dorfberger S., Adi-Japha E., Karni A. (2009). Sex differences in motor performance and motor learning in children and adolescents: An increasing male advantage in motor learning and consolidation phase gains. Behav. Brain Res..

[12] Barnett L.M., van Beurden E., Morgan P.J., Brooks L.O., Beard J.R. (2010). Gender Differences in Motor Skill Proficiency From Childhood to Adolescence. Res. Quart. Exerc. Sport.

[13] O’Brien-Smith J., Bennett K.J.M., Fransen J., Smith M.R. (2020). Same or different? A comparison of anthropometry, physical fitness and perceptual motor characteristics in male and female youth soccer players. Sci. Med. Football.

[14] Perroni F., Gallotta M.C., Pisano S., Reis V.M., Emerenziani G.P., Guidetti L., Baldari C. (2018). Gender differences in anthropometric parameters and technical performance of youth soccer players. Sport Sci. Health.

[15] Peterson M., Alvar B., Rhea M. (2006). The Contribution of Maximal Force Production to Explosive Movement Among Young Collegiate Athletes. J. Strength Cond. Res..

[16] Nunome H., Drust B., Dawson B. (2013). Analysis of Finnish young soccer players’ passing and dribbling skills. Science and Football VII.

[17] Ali A., Foskett A., Gant N. (2008). Validation of a Soccer Skill Test for Use with Females. Int. J. Sports Med..

[18] Ali A., Williams C., Hulse M., Strudwick A., Reddin J., Howarth L., Eldred J., Hirst M., McGregor S. (2007). Reliability and validity of two tests of soccer skill. J. Sports Sci..

[19] Mujika I., Santisteban J., Impellizzeri F.M., Castagna C. (2009). Fitness determinants of success in men’s and women’s football. J. Sports Sci..

[20] Thomas J., French K. (1985). Gender Differences Across Age in Motor Performance. A Meta-Analysis. Psychol. Bullet.

[21] Liu H., Gómez M.-A., Gonçalves B., Sampaio J. (2016). Technical performance and match-to-match variation in elite football teams. J. Sports Sci..

[22] Russell M., Benton D., Kingsley M. (2010). Reliability and construct validity of soccer skills tests that measure passing, shooting, and dribbling. J. Sports Sci..

[23] Bennett K., Novak A., Pluss M., Coutts A., Fransen J. (2018). Assessing the validity of a video-based decision-making assessment for talent identification in youth soccer. J. Sci. Med. Sport.

[24] Pedersen A.V., Lorås H., Norvang O.P., Asplund J. (2014). Measuring soccer technique with easy-to-administer field task in female soccer players from four different comeptitive levels. Perc. Mot. Skills.

[25] Sørensen A., Sørensen V., Dalen T. (2021). A Novel Approach for Comparison of Reception Performance in a Technique Test and Small-Sided Games. Sports.

[26] Kelly A., Wilson M.R., Jackson D.T., Williams C.A. (2020). Technical testing and match analysis statistics as part of the talent development process in an English football academy. Int. J. Perf. Anal. Sport.

[27] Cohen J. (1988). Statistical Power Analysis for the Behavioral Sciences.

[28] Ford P.R., Ward P., Hodges N.J., Williams A.M. (2009). The role of deliberate practice and play in career progression in sport: The early engagement hypothesis. High Abil. Stud..

[29] Erikstad M.K., Høigaard R., Johansen B.T., Kandala N.-B., Haugen T. (2018). Childhood football play and practice in relation to self-regulation and national team selection; a study of Norwegian elite youth players. J. Sports Sci..

[30] Sharkey B.J., Gaskill S.J. (2006). Sport Physiology for Coaches.

[31] Trost S., Pate R., Dowda M., Saunders R., Felton G. (1996). Gender Differences in Physical Activity and Determinants of Physical Activity in Rural Fifth Grade Children. J. School Health.

[32] Owen A., Wong D.P., Paul D., Dellal A. (2014). Physical and Technical Comparisons between Various-Sided Games within Professional Soccer. Int. J. Sports Med..

[33] Magill R.A., Anderson D.I. (2017). Motor Learning and Control: Concepts and Applications.

[34] Pedersen A.V., Lorås H. (2017). When Is a Test Score Fair for the Individual Who Is Being Tested? Effects of Different Scoring Procedures across Multiple Attempts When Testing a Motor Skill Task. Front. Psychol..

